# Autosomal *gsdf* acts as a male sex initiator in the fish medaka

**DOI:** 10.1038/srep19738

**Published:** 2016-01-27

**Authors:** Xi Zhang, Guijun Guan, Mingyou Li, Feng Zhu, Qizhi Liu, Kiyoshi Naruse, Amaury Herpin, Yoshitaka Nagahama, Jiale Li, Yunhan Hong

**Affiliations:** 1 Department of Biological Sciences, National University of Singapore, 14 Science Drive 4, Singapore 117543, Singapore; 2 Key Laboratory of Exploration and Utilization of Aquatic Genetic Resources of Ministry of Education and College of Fisheries and Life Sciences, Shanghai Ocean University, Shanghai 201306, China; 3 Laboratory of BioResources, National Institute for Basic Biology, Okazaki, Aichi 444-8585, Japan; 4 INRA, UR1037 Fish Physiology and Genomics, Rennes F-35000, France; 5 South Ehime Fisheries Research Center, Ehime University, Matsuyama 790-8577, Japan

## Abstract

Sex is pivotal for reproduction, healthcare and evolution. In the fish medaka, the Y-chromosomal *dmy* (also *dmrt1bY*) serves the sex determiner, which activates *dmrt1* for male sex maintenance. However, how *dmy* makes the male decision via initiating testicular differentiation has remained unknown. Here we report that autosomal *gsdf* serves a male sex initiator. Gene addition and deletion revealed that *gsdf* was necessary and sufficient for maleness via initiating testicular differentiation. We show that *gsdf* transcription is activated directly by *dmy*. These results establish the autosomal *gsdf* as the first male sex initiator. We propose that *dmy* determines maleness through activating *gsdf* and *dmrt1* without its own participation in developmental processes of sex initiation and maintenance. *gsdf* may easily become a sex determiner or other autosomal genes can be recruited as new sex determiners to initiate *gsdf* expression. Our findings offer new insights into molecular mechanisms underlying sex development and evolution of sex-controlling genes in vertebrates.

The majority of animal species have both sexes, male and female, for sexual reproduction. Sex development is a multi-step process including sex determination, initiation, differentiation and maintenance, culminating in the production of sperm or eggs for germline transmission. Defects in each of these steps may lead to sex abnormalities including infertility and sex reversal. Animal sex control has an impact in animal husbandry and even human healthcare^1^. Sex is determined by environmental and genetic factors^2^. A hallmark of genetic mechanisms underlying sex determination and development is that they show remarkable diversity and do not follow the evolutionary history, because they can arise independently and rapidly, leading to enormous variation between even closely related species. For example, even master sex-determining genes or sex determiners (SDs) so far identified in different animal taxa show considerable differences in sequence and activity of their products^3^. The primary role of an SD is to determine the initial sex by triggering testicular or ovarian differentiation of a sexually bi-potential gonad. The presence of an SD determines the genetic sex, whereas the onset of gonadal differentiation towards a testis or an ovary delineates primary sex initiation. SDs act at the top of hierarchical cascades to control sex differentiation^4^. For example, *sry* in mammals initiates testicular differentiation through activating its direct target *sox9*^5^,^6^. Notably, the cascades downstream of SDs also vary enormously from one animal to another^4^.

The enormous diversity of genetic sex determination mechanisms is a long-standing mystery and also a major challenge for understanding sex development^2^. Fish have sex-determination mechanisms ranging from environmental to different modes of genetic determination and thus provide a paradigm for studying sex plasticity and development. In particular, medaka (*Oryzias latipes*) is an excellent vertebrate model for sex development^7^ and reproductive biotechnology^8^,^9^. This fish is the first vertebrate that showed crossing-over between X and Y chromosomes^10^, induction of sex reversal^11^, and most importantly, offered the first vertebrate SD besides the mammalian *sry*, namely *dmy*^12^, *dmrt1y*^13^ or *dmrt1bY*^14^. Most recent studies have revealed female germ stem cell markers capable of making intrinsic sperm-egg fate decision in medaka^15^. It is known that *dmy* activates *dmrt1*^16^, which in turn maintains testicular differentiation, as *dmrt1* mutation causes male-to-female sex reversal after the initiation of testicular differentiation^17^. However, how *dmy* exerts its primary role in male decision via triggering testicular differentiation has remained unknown. Paradoxically, there are several cases where *dmy* is dispensable for maleness^13^,^18^,^19^, which points to the presence of autosomal gene(s) essential for male sex initiation in this organism^14^,^18^,^20^.

Recently, the gene *gsdf* is emerging as a novel sex related factor in several distantly related fish species. This teleost-specific gene^21^ encodes the gonadal soma derived factor, which belongs to the transforming growth factor-β superfamily^22^. In medaka, *gsdf* is located on chromosome 12 and is predominantly expressed in the Sertoli cells and granulosa cells in mature gonads^23^. Here we show this autosomal gene *gsdf* acts as a male sex initiator downstream of *dmy* and renders itself a prime candidate for the searched autosomal gene essential for maleness.

## Results

### *gsdf* addition causes masculinization

**Figure 1:**
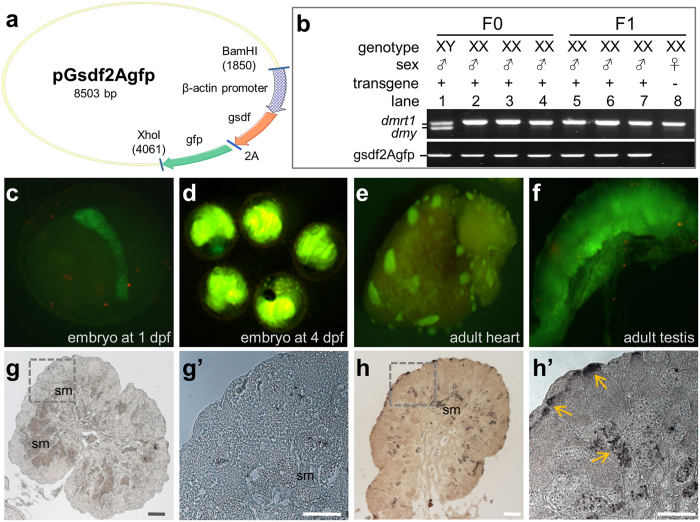
Masculinization by ectopic g*sdf* expression. (**a)** Map of pGsdf2Agfp. The 2.5 kb *β-actin* promoter, *gsdf*, *gfp* and self-cleavage sequence *2A* as well as flanking restriction sites are indicated. (**b)** Genotype and phenotype of F0 adults. (**c–f)** Transgenic GFP expression in F1 embryos at 1 dpf (**c**) or 4 dpf (**d**) and adult heart (**e**) and testis (**f**). (**g,h)** Cross sections of adult gonads immunostained with an anti-GFP antibody, showing testicular structure and presence of many sperm of a wildtype XY gonad (**g**) and a XX gonad transgenic for pGsdf2Agfp. (**g’,h’)** Larger magnification of areas framed in (**g**) and (**h**), highlighting the absence in (**g’**) and presence in (h’) of GFP (arrows). sm, sperm. Scale bar, 50 μm.

**Figure 2:**
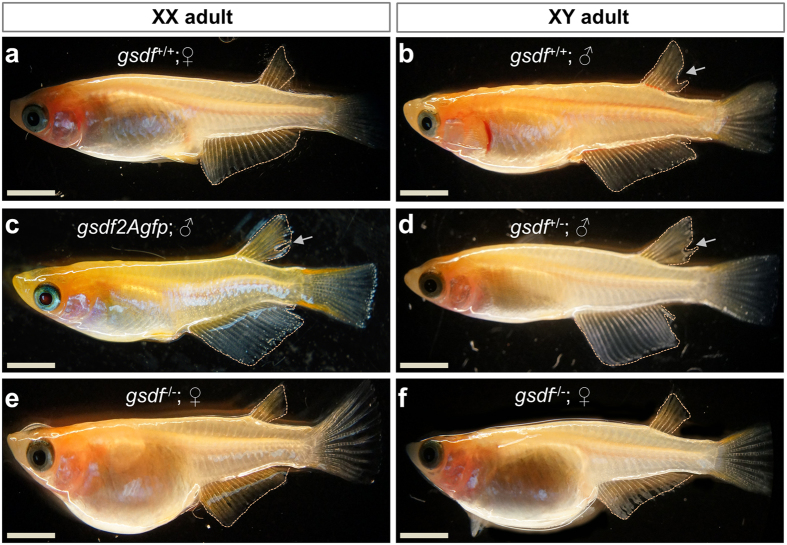
Sexual phenotype of medaka adults. **(a)** wtXX female, showing a triangular-shaped anal fin and connected dorsal fin rays. (**b)** wtXY male, showing a parallelogram-shaped anal fin and the separation (arrow) of hindmost dorsal fin rays. (**c)** Male phenotype of *gsdf2Agfp*-transgenic XX fish. (**d)** Male phenotype of *gsdf*^+/−^ XY fish. (**e)** Female phenotype of mtXX fish, showing dramatically enlarged abdomen. (**f)** Female phenotype of mtXY fish, showing dramatically enlarged abdomen. mt, homozygous *gsdf*^*−/−*^mutant; wt, wildtype. Scale bar, 0.5 cm.

It has been reported that two genes are sufficient to induce medaka masculinization upon transgenic addition, one is *dmy*^24^ and the other is *gsdf* ^*Y*^, a Y-chromosomal copy of *gsdf* acting as the SD in *O. luzonesis*^25^. We asked whether the medaka autosomal *gsdf* was similar to *gsdf* ^*Y*^ in function. To this end, pGsdf2Agfp expressing the Gsdf and GFP from the *β*-actin promoter was constructed (Fig. 1a) and microinjected into 174 early medaka embryos at the 1-cell stage. Out of 108 adults obtained, eight XY and nine XX fish contained the transgene (Table 1) as determined by genotyping (lanes 1–4; Fig. 1b). While all F0 adults without the transgene detected in the caudal fin developed to either females or males in accordance with their genotype, five of the nine transgenic XX adults were found to be males in phenotype, producing a 56% masculinization rate in F0 animals. Three of them (lanes 2–4, Fig. 1b) were fertile and used to mate with normal XX females. A total of 219 F1 adults were analyzed, all of them were XX as expected from the mating scheme (Table 1). We obtained a total of 68 transgenic adults in F1 generation from the three founders (48, 14 and 6 respectively). Transgene expression was observed ubiquitously - as expected for the promoter used - in developing embryos (Fig. 1c,d) and adult organs such as the heart (Fig. 1e) and testis (Fig. 1f). Notably, GFP-expressing gonads in XX adults were mature testes (Fig. 1f). On the gonadal sections immunostained with an anti-GFP antibody, a normal XY fish displayed a typical architecture of a mature testis containing many sperm but lacked GFP staining (Fig. 1g,g’), whereas a *gsdf*-transgenic XX fish did show strong GFP staining and the absence of detectable female germ cells but the presence of a testicular structure and many sperm (Fig. 1h,h’). Medaka adults display easily distinguishable secondary sex characteristics^26^. Specifically, the hindmost rays are separated from other rays in the dorsal fin in male but linked together in female, and the anal fin is parallelogram-shaped in male but triangular-shaped in female (Fig. 2a,b). All the 68 transgenic fish invariantly displayed typical male secondary sex characteristics (Fig. 2c), producing a 100% efficiency for masculinization. Taken together, *gsdf* is fully comparable to SDs, *dmy* and *gsdf*^*Y*^, in the ability to cause masculinization of genetic females at gonadal, gamete and organismal levels.

### *gsdf* deletion causes feminization

In order to determine the exact role of *gsdf* in maleness, we then analyzed the *gsdf* deletion phenotype. Several fish bearing subtle mutations in the *gsdf* locus were obtained by using zinc finger nucleases (ZFN) (Supplementary Fig. 1a,b)^27^. A male founder carrying a null *gsdf* allele was chosen to produce a mutant family. This allele contained a 4-bp insertion and predicted truncated translation (Supplementary Fig. 1c). Upon crossing with wildtype females, this fish produced 80 F1 adults, five of which had the null *gsdf* allele and developed into XX females (n = 3) and XY males (n = 2). Intercrossing between F1 heterozygotes (*gsdf*^+/−^) produced 120 F2 adults and a typical Mendelian segregation of wildtype (n = 35; *gsdf*^+/+^), *gsdf*^+/−^ (n = 62) and homozygous animals (n = 23; *gsdf*^*−/−*^) (Table 2). *gsdf* disruption led to multiple phenotypes in adulthood, and the most prominent was feminization, which was the focus of this study. All the heterozygotes developed as female (n = 25) or male (n = 37) strictly according to their genetic sex (Fig. 2d). Interestingly, all the 23 *gsdf*^*−/−*^ adults including 13 fish of the XY chromosomal constitution were found to be female (Fig. 2e,f). The *gsdf*^+/−^ F2 fish were selected to produce the progeny and the Mendelian segregation also occurred in the resultant F3 generation, where all *gsdf*^+/−^ fish (n = 129) again developed according to genetic sex, whereas *gsdf*^*−/−*^ adults (n = 56; 19 XX and 37 XY) were invariantly female in phenotype (Table 2). The Mendelian segregation in F2 and F3 generations and the fertility of phenotypically female *gsdf*^*−/−*^ XY adults demonstrated that *gsdf* is dispensable for survival, and its disruption has caused 100% feminization at the gamete and organismal levels.

**Figure 3:**
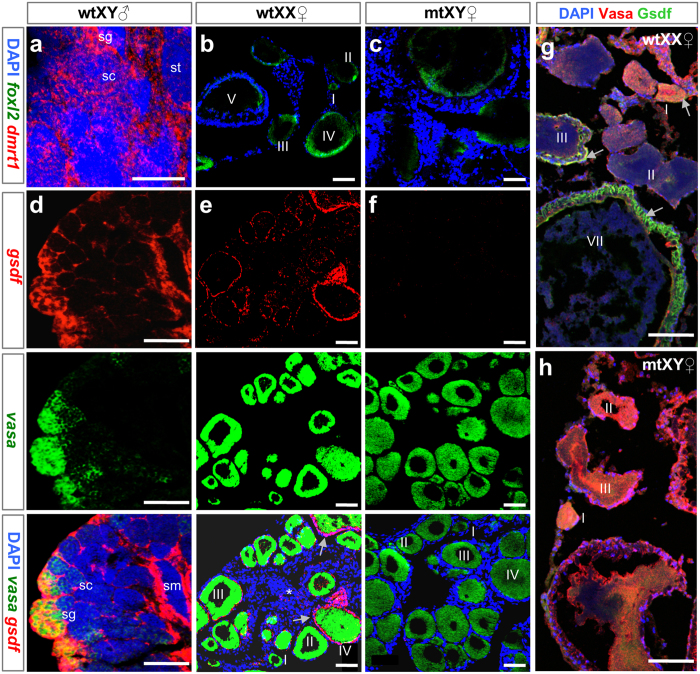
Gonadal histology and gene expression. Cryosections of adult gonads were subjected for FISH with antisense riboprobes of *dmrt1* plus *foxl2*, *gsdf* plus *vasa* or IF with antibodies against Vasa plus Gsdf and analyzed by fluorescence microscopy. (**a–c)** FISH analysis, showing the expression of *dmrt1/dmy* (red) and *foxl2* (green) transcripts. (**d–f)** FISH analysis. The *gsdf* RNA signal is evident in somatic cells surrounding spermatogonia (sg), spermatocytes (sc) and sperm (sm) of the wtXY testis (**d**), somatic cells surrounding (arrows) oocytes of various stages (I-IV) and residing in the interstitium (asterisk) of the wtXX ovary (e), but barely detectable in the mtXY gonad (**f**). (**g,h)** IF analysis, showing the expression of Vasa (red) and Gsdf protein (green). The Gsdf protein is seen in somatic cells surrounding (arrows) oocytes of the wtXX ovary (**g**) but absent in the mtXY gonad (**h**). mt, homozygous *gsdf*^*−/−*^mutant; wt, wildtype. Scale bar, 50 μm.

We examined the histology of adult gonads and spatial gene expression patterns by fluorescence *in situ* hybridization (FISH) and immunofluorescence (IF). We first focused on *dmrt1* and *foxl2*. The former is expressed in Sertoli cells and essential for the maintenance of testicular differentiation^17^. The latter is predominantly expressed in the granulosa cells of the medaka ovary^28^. The *dmrt1* riboprobe hybridized to the *dmy* transcripts due to sequence similarity^29^. On the gonadal sections, the *dmrt1* or *dmy* transcripts were evident in the Sertoli cells surrounding spermatogonia, spermatocytes and sperm of the wildtype XY (wtXY) testis (Fig. 3a) but hardly detectable in the wildtype XX (wtXX) ovary (Fig. 3b). In the *gsdf*-mutant XY (mtXY) gonad, the *dmrt1* riboprobe produced a barely detectable signal (Fig. 3c), possibly due to its cross hybridization to the *dmy* transcripts. On the other hand, the *foxl2* mRNA was absent in wtXY testis (Fig. 3a) but present in follicular cells of the wtXX ovary and more importantly, the mtXY gonad (Fig. 3b,c), suggesting that the mtXY gonad is comparable to the wtXX ovary in architecture and gene expression. We then analyzed the expression of *vasa* and *gsdf*. *vasa* was expressed predominantly in spermatogonia of adult testes (Fig. 3d) and pre-meiotic oocytes of adult ovaries from wtXX fish and mtXY fish (Fig. 3e,f), in accordance with its reported expression pattern^30^,^31^. The *gsdf* signal peaked in putative Sertoli cells surrounding spermatogonia and sperm, remained easily detectable in putative Sertoli cells surrounding spermatocytes of the wtXY testis (Fig. 3d), and was moderately detectable in the somatic cells surrounding developing oocytes and those residing in the interstitium of the wtXX ovary (Fig. 3e), which is consistent with its reported expression pattern^23^. Notably, the *gsdf* signal was hardly detectable in the mtXY gonad (Fig. 3f). IF by using an anti-Gsdf antibody (αGsdf) revealed the localization of Gsdf protein also in somatic cells surrounding developing oocytes of the wtXX ovary (Fig. 3g). Gsdf protein was, however, completely absent in the mtXY gonad (Fig. 3h), demonstrating that the mutation by 4-bp insertion leads to *gsdf* disruption at RNA, protein and thus functional levels. Therefore, *gsdf* disruption causes full feminization of an XY gonad into a functional ovary.

### *gsdf* disruption alters global gene expression

**Figure 4:**
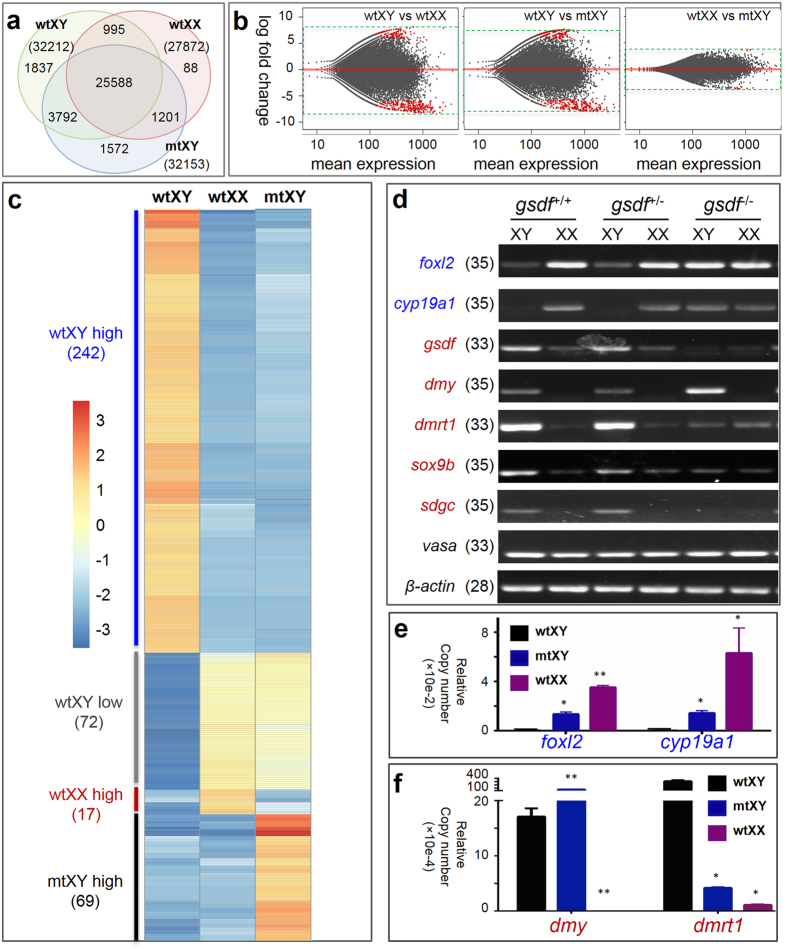
Gene expression profile in adult gonads. (**a)** Transcriptome analysis. Venn diagram, showing numbers of unigenes expressed in wtXY, wtXX and mtXY gonads. (**b)** MA-plots, showing the comparisons of global gene expression profiles. Each gene is represented as a dot. Red dots, representing significantly differentially expressed genes (P < 0.05); y-axis, the log2 fold change; x-axis, the average of counts (mean expression). (**c)** Heat map of 400 top differentially expressed genes. (**d)** RT-PCR analysis, showing expression of ovarian (blue) and testicular markers (red). vasa and *β-actin* served a germ cell marker and a loading control. Numbers of PCR cycles are indicated in the parenthesis. (**e,f)** ddPCR analyses, showing the relative mRNA levels of ovarian markers (**e**) and testicular markers (**f**). Values are relative copy numbers per μl of ddPCR templates normalized to *β-actin* and presented as means ± s.e.m from three independent experiments. Significance levels of difference (*P < 0.05; **P < 0.01) were obtained by comparisons to wtXY as the control.

We analyzed global gene expression profiles to gain insights into the molecular mechanism underlying feminization upon *gsdf* disruption. Next-generation sequencing of adult gonads of from wtXY, wtXX and mtXY fish produced a total of 35073 unigenes, 25588 of which were widely expressed in gonads of the three genotypes. The wtXX ovary and wtXY testis had 27872 and 32212 unigenes, respectively. The mtXY gonad generated 32153 unigenes, indicating that it is not different from the wtXY testis in total number of expressed unigenes (Fig. 4a). However, a comparison of globally differential gene expression profile in log fold change revealed the mtXY gonad is more similar to the wildtype ovary than to the wildtype testis (Fig. 4b). This similarity became more evident when a comparison was focused on 400 most differentially expressed genes, where 314 unigenes (78.5%) were expressed at either low (n = 242) or high levels (n = 72) in wtXX and mtXY gonads compared to the wtXY testis (Fig. 4c). Interestingly, 17 and 69 unigenes were highly expressed only in the wtXX and mtXY gonads, respectively (Fig. 4c). Thus, *gsdf* disruption alters global gene expression in favor of ovarian development.

We then examined the gonadal expression profile of genes as markers or essential players for female and male development. Genes chosen were 2 ovarian markers *foxl2* and *cyp19a1* (*aromatase*)^28^,^32^ and 5 testicular markers *gsdf*, *dmy*^12^,^13^, *sdgc*, *dmrt1* and *sox9b*. *sdgc* is linked closely to *dmy* and expressed predominantly in spermatogonia but weakly in early oocytes^33^. *dmrt1* and *sox9b* are autosomal male markers, as *dmrt1* is expressed in the Sertoli cell lineage and essential for the maintenance of testis differentiation^17^; while *sox9b* is expressed in both sexes during early gonadal sex differentiation^34^ but maintained mainly in the XY gonads later during testicular tubules development^35^. *vasa* was used as a germ cell marker^30^,^36^. An RT-PCR analysis revealed that in the mtXY gonad, both ovarian markers *foxl2* and *cyp19a1* were dramatically increased to a level comparable to that in the wtXX ovary, whereas testicular markers *dmrt1* and *sox9b* were concurrently decreased obviously (Fig. 4d). Notably, the mutant *gsdf* RNA became hardly detectable in both XX and XY gonads (Fig. 4d), in accordance with reported decay of nonsense transcripts^37^. Interestingly, *dmy* was significantly up-regulated in the mtXY gonad, perhaps due to a decreased level of *dmrt1* capable of suppressing *dmy* expression^16^. More importantly, the *sdgc* RNA was barely detectable in the mtXY gonad, indicating the absence of spermatogonia (Fig. 4d). A quantitative droplet digital PCR (ddPCR) analysis validated the RT-PCR results. Specifically, the expression of *foxl2* and *cyp19a1* in the mtXY gonad was approximately 3 times lower than in the wtXX ovary, but significantly higher than in the wtXY testis (Fig. 4e). The expression of *dmrt1* was dramatically downregulated in the mtXY gonad than in the wtXY testis (Fig. 4f), indicating that *gsdf* may directly or indirectly activate or maintain its expression. Interestingly, *dmy* was significantly upregulated in the mtXY gonad than in the wtXY testis, demonstrating that *gsdf* is not necessary for *dmy* expression. Thus, *gsdf* disruption causes gonadal feminization at the molecular level, and *gsdf* may play an important role also in regulating gene expression favoring testicular versus ovarian development and function.

### Feminization via ovarian differentiation

**Figure 5:**
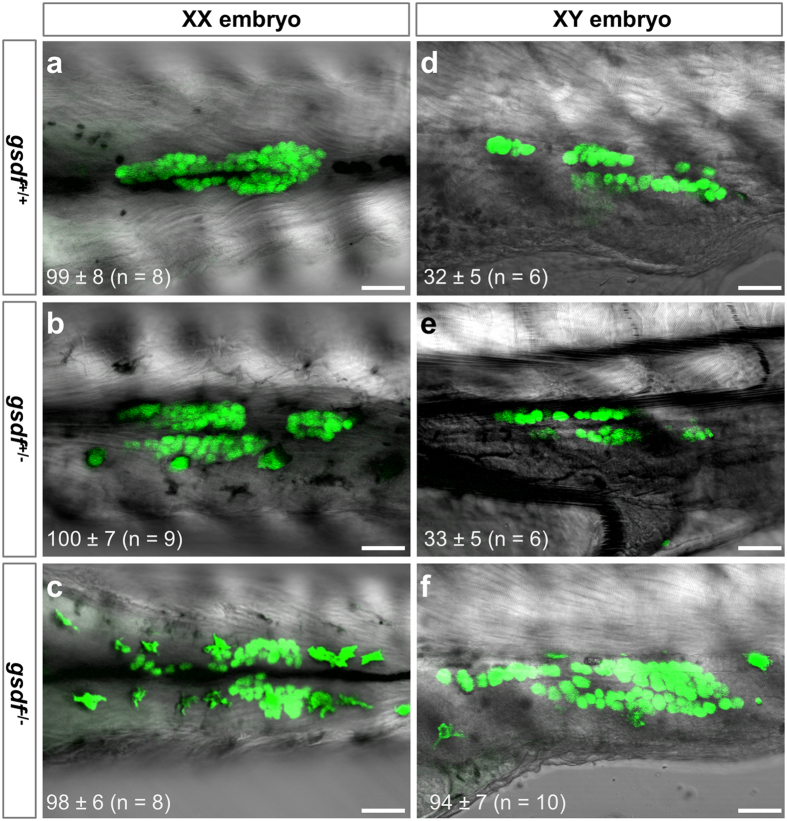
PGC abundance at early sexual differentiation. Hatching F3 embryos (9 dpf) from crosses between *gsdf*^+/−^ fish and *Vg* fish bearing GFP-labeled germ cells were analyzed by confocal microscopy and then genotyped. GFP-positive PGCs (green) are clearly visible in the gonad. (**a–c)**, XX gonads, showing similar abundance of PGCs in normal (**a**), *gsdf*^+/−^ (**b**) and *gsdf*^*−/−*^ (**c**) embryos. (**d–f)**, XY gonads, highlighting fewer PGCs in normal (**d**) and *gsdf*^+/−^ embryos (**e**) than XX embryos (**a–c**), whereas the PGC number in *gsdf*^*−/−*^ embryos (**f**) is comparable to that in XX embryos (**a–c**). Numbers of PGCs are presented as means ± SD with number of embryos examined are given in parenthesis. Scale bar, 50 μm.

In order to distinguish whether feminization upon *gsdf* disruption comes from either male-to-female sex reversal after the initiation of testicular differentiation or ovarian differentiation without the initiation of testicular differentiation in genetically male embryos, we determined the initial primary sex of differentiating gonads. In medaka, sex dimorphism is easily detectable in embryos shortly before and after hatching, when primordial germ cells (PGCs) in a male gonad are entering into mitotic arrest and thus dramatically fewer than in its female counterpart, where germ cells continue propagation and enter into meiosis^29^,^38^. To visualize PGCs in live embryos, a *gsdf*^+/−^ male was crossed to a *Vg* female homozygous for transgene Ol*vasa-gfp* that expresses GFP specifically in germ cells, and embryos with GFP-labeled PGCs from the F3 generation were used for PGC observation (Supplementary Fig. 3). As expected, these F3 embryos had various genotypes as determined by genomic PCR genotyping. These embryos at stage 39 (1 day before hatching) displayed two major types in terms of PGC number, namely the female type of more PGCs and male type of fewer PGCs. XX gonads had a similarly larger PGC number (~100) in wildtype (n = 8), *gsdf*^+/−^ (n = 9) and *gsdf*^*−/−*^ (n = 8) embryos (Fig. 5a–c), which was characteristic of ovarian differentiation and in accordance with a female phenotype in adulthood (Fig. 2a,e). In contrast, XY gonads had a similarly smaller PGC number (~30) in wildtype (n = 6) and *gsdf*^+/−^ (n = 6) embryos (Fig. 5d,e), which was in accordance with testicular differentiation and a male phenotype in adulthood (Fig. 2b,d). Strikingly, the number of PGCs in mtXY gonads examined (n = 10) increased to ~90 (Fig. 5f) and was in accordance with ovarian differentiation as in XX gonads. Taken together, feminization upon *gsdf* disruption is not a consequence of male-to-female sex-reversal but rather results from ovarian differentiation in the absence of testicular differentiation, demonstrating that *gsdf* is a male sex initiator, because it is necessary for the initiation of testicular differentiation, the first step of male development.

### *gsdf* acts directly downstream of *dmy*

**Figure 6:**
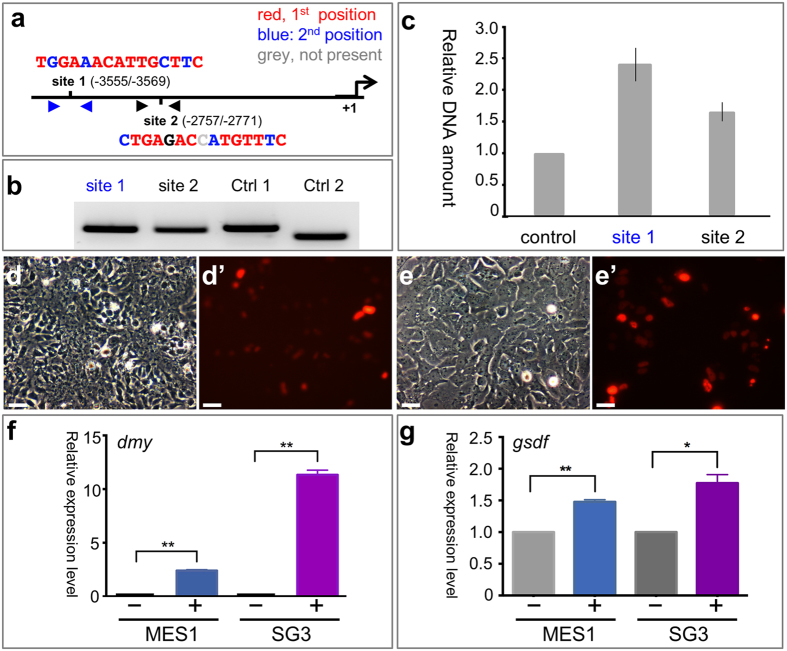
*gsdf* is a direct target of *dmy*. **(a)** Dmy target sites. Shown are the positions and sequences of two putative Dmy binding sites within the 4-kb medaka *gsdf* promoter as well as the transcription start point (+1). Bases in red and blue or grey match the most and second most frequent bases or do not match in respective positions of the reported matrix. (**b)** Specificity of PCR-based assay. (**c)** ChIP assay, showing PCR products from immunoprecipitated DNA fragments. Notably, significant binding by Dmy is clearly seen for both binding site 1 and site 2 at P < 0.01. The immunoprecipitated DNA fragments of the control group were obtained from ΔDmy::GFP-transgenic fish (without the DNA binding domain, DM) by the same anti-GFP antibody. Controls show no enrichment while both sites reveal nearly two-fold enrichment of binding by dmy::GFP. (**d–e’)** Phenotype of medaka stem cell lines MES1 (**d,d’**) and SG3 (**e,e’**) at 72 h post transfection with pDmy:cherry. Scale bar, 20 μm. (**f–g)** ddPCR analysis of *dmy* (**f**) and *gsdf* (**g**) expression. −, control cells; +, sorted cells positive for transgene-expressed Cherry and thus Dmy. A nearly 2-fold increase in *gsdf* RNA is clearly seen in both MES1 and SG3 cell lines by transgenic *dmy* expression (**g**). *dmy* expression was presented as relative copy numbers per μl of ddPCR templates normalized to *β-actin* and presented as means ± s.e.m from three independent experiments. *gsdf* expression was presented as relative gene expression levels to the controls and presented as means ± s.e.m from three independent experiments. *P < 0.05; **P < 0.01 from comparisons between transgenic and non-transgenic control cells.

The experiments described so far demonstrate that *gsdf* is essential for early testicular differentiation, a role similar to *dmy*^12^,^13^. This similarity provoked us to investigate whether *dmy* and *gsdf* had direct or indirect relationship in action. Two putative binding sites of Dmy are present within the 4-kb *gsdf* upstream regulatory region (Fig. 6a) and can be specifically amplified for quantification (Fig. 6b). In the testis of medaka transgenic for dmy:GFP (also dmrt1bY:GFP)^16^, both sites showed approximately 2-fold enrichment of binding by dmy:GFP as determined by Chromatin immunoprecipitation (ChIP) using a GFP antibody (Fig. 6c). An *in vitro* reporter assay was performed to see whether *dmy* controls *gsdf* transcription in medaka embryonic stem cell line MES1^39^ and spermatogonial cell line SG3^8^. Both cell lines were transfected with pDmy:cherry and cells expressing the Dmy:Cherry fusion protein were not different from nontransgenic control cells in phenotype (Fig. 6d–e’ and Supplementary Fig. 4a,b). Transgenic cells were isolated by fluorescence-activated cell sorting (Supplementary Fig. 4c) and analyzed for altered *dmy* and *gsdf* RNA expression. Both cell lines lacked a detectable level of *dmy* transcripts (Fig. 6f) but a low level of *gsdf* transcripts (Fig. 6g). As expected, *dmy* was expressed at a high level in the sorted transgenic cells (Fig. 6f), where a nearly 2-fold increase in the *gsdf* RNA level was also observed (Fig. 6g). Taken together, *gsdf* is a direct target of *dmy* for transcriptional activation and conforms to its genetic hypostasis to *dmy* in action.

## Discussion

Previously we and others have established *dmy* as the male SD in medaka, as it is the only functional gene within the 258-kb region unique to the Y chromosome^13^,^40^ and shows necessity^12^ and sufficiency^24^ for maleness. However, *dmy* is not a sufficient factor to ensure the maleness and may also be paradoxically dispensable for maleness in several cases. For example, medaka XY embryos can develop into functional females after estrogen treatment in the presence of *dmy* expression^13^, and XX embryos can develop into functional males after treatment with either androgen^41^,^42^ or high temperature^43^,^44^ in the absence of *dmy*. Notably, *dmy* expression is independent of sex phenotype and remains unaltered by feminizing^13^ and masculinizing^29^ factors^45^. In addition, medaka shows a strain difference in frequency of spontaneous XX males^18^. The fact that *dmy* is not always necessary and sufficient for maleness is obviously in consistence with an important notion: *dmy* acts upstream in the genetic hierarchy^13^ and serves as the male SD only, whereas autosomal genes are responsible for male sex differentiation and maintenance^13^,^14^,^18^,^20^. Indeed, autosomal *dmrt1* behaves as a male sex maintainer, because its mutation leads to male-to-female sex reversal after the initiation of testicular differentiation^17^. However, its addition did not masculinize genetic females^24^ apparently due to its inability to induce testicular initiation or differentiation, as its expression is not detectable until 20 days post hatching after testicular differentiation^13^,^29^ via transcriptional activation by *dmy*^16^ and can be elevated by masculinizing factors like high temperature^46^.

While this manuscript was under revision, another group also reported that *gsdf* mutation has induced XY gonads to undergo ovarian differentiation, suggesting its potential role as an endogenous inducer of testicular development^47^. Here our study has provided sufficient evidence and established *gsdf* as a male sex initiator acting downstream of *dmy*, thus serving as a prime candidate for the searched autosomal gene essential for maleness. First, *gsdf* addition is sufficient for masculinization in the absence of *dmy*, and *gsdf* disruption causes feminization without compromising *dmy* expression, pointing to its hypostasis to *dmy* in action. Second, feminization upon *gsdf* disruption results from the initiation of ovarian differentiation despite the presence of *dmy*, demonstrating that the earliest and perhaps the primary role of *gsdf* is to initiate testicular differentiation, which cannot be replaced by *dmy*^13^. Finally, the fact that Dmy protein binds to the *gsdf* promoter and activates *gsdf* transcription convincingly reveals that *gsdf* acts downstream of *dmy*. Consistent with this notion is a recent report that the *gsdf* mRNA reduces dramatically by up to 28 folds in the *dmy* knockout XY gonad^48^. Reportedly, *gsdf* expression temporospatially correlates with the initiation of testicular differentiation and is suppressed by feminizing factors such as female hormone estrogen^23^ but activated by masculinizing factors such as male hormone androgen and high temperature^49^. This expression pattern is what exactly expected for a male sex initiator. Interestingly, unlike the recent report showing that some genetically male adults did not demonstrate female phenotype after *gsdf* disruption^47^, here we have revealed a 100% feminization rate of XY adults, indicating that the adult sexual phenotype might be affected by multiple factors. Moreover, *gsdf* disruption causes the alteration of global gene expression and *dmrt1* down-regulation in adult gonads, suggesting the potential involvement of *gsdf* in subsequent processes such as male sex maintenance besides acting as a male sex initiator.

**Figure 7:**
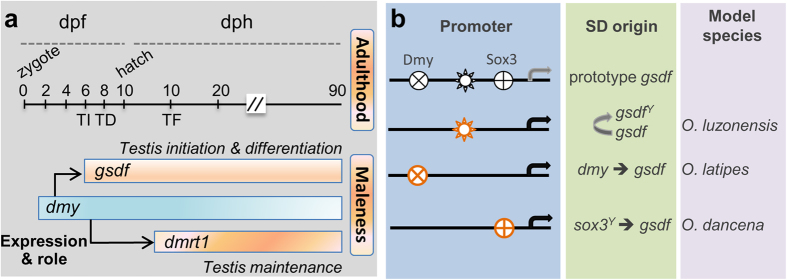
Sex determination and its evolution in *Oryzias*. **(a**) The core cascade controlling sex development in medaka. The black horizontal line depicts developmental day(s) post fertilization (dpf) or hatching (dph). Shown are key developmental events including testis initiation (TI), testis differentiation (TD) and testis formation (TF). Horizontal boxes depict developmental RNA expression stages of *dmy*, *gsdf* and *dmrt1*. Arrows depict transcriptional activation of *gsdf* and *dmrt1* by *dmy*. Indicated are the primary roles for *gsdf* in testis initiation and differentiation and for *dmrt1* in testis maintenance. (**b)** Hypothetical evolution of sex determiners in the genus *Oryzias*. *gsdf* may easily become a sex determiner (namely *gsdf*^*Y*^ in *O. luzonesis*) via acquiring proper temporospatial expression (bent arrow) or preferentially recruit *gsdf*-regulating genes as new sex determiners (namely *dmy* in *O. latipes* and *sox3*^*Y*^ in *O. dancena*). Shown are potential or putative binding sites (black) for Dmrt1, Sox3 and an unknown transcription factor in the prototype *gsdf* promoter. These binding sites may be used (orange) by transcription factors such as Dmrt1 and Sox3 for activation (horizontal arrows) of proper temporospatial *gsdf* expression to initiate testicular differentiation.

In model organisms such as *Drosophila* and *C. elegans*^4^, a single genetic cascade responsible for sex determination and development comprises multiple genes. This study together with previous reports reveals two unusual features for the genetic male sex determination system in medaka, where *dmy*, *gsdf* and *dmrt1* constitute the core sex determination system and form two cascades. One comprises *dmy* and *gsdf* for male sex initiation and differentiation, the other comprises *dmy* and *dmrt1* for male sex maintenance (Fig. 7a). As mentioned above, *gsdf* and *dmrt1* are involved in male sex initiation and maintenance, whereas *dmy* itself is not necessary for, and apparently not involved in, these two early developmental processes. We propose that *dmy* determines maleness through activating the male sex initiator *gsdf* and the male sex maintainer *dmrt1* without its own participation in developmental processes of sex initiation and maintenance.

SDs demonstrate enormous diversity across animal phyla in general as well as conservation within certain taxa such as *sry* in many mammals and *transformer* (*tra*) in several insects^2^. There is an example that certain genes such as *dmrt1* or its paralog can be repeatedly recruited as the SD in a wide variety of organisms including medaka^12^, smooth tongue sole (*Cynoglossus semilaevis*)^50^, chicken^51^ and frog^52^. These facts raise a question as to what genes can preferentially become SDs. Our identification of autosomal *gsdf* as a male sex initiator has a direct and important implication on the evolution of SDs as well as sex chromosomes. It is generally accepted that autosomes bearing genes capable of exerting a male determining function may evolve into Y chromosomes that will compete and possibly replace the present Y chromosome^14^, ultimately leading to the tremendous variety of SDs in lower vertebrates due to the plasticity of homomorphic sex chromosomes^53^. Here we propose that autosomal genes performing key male sex functions like *gsdf* may serve as a core in understanding the evolution of SDs and cascades (Fig. 7b). On one hand, *gsdf* may easily evolve into a male SD capable of competing and even replacing the existing SD *dmy*, which is exactly what has occurred in *O. luzonesis* (Fig. 7b), where *gsdf* has undergone allelic divergence into *gsdf*^*Y*^ and *gsdf*^*X*^, with *gsdf*^*Y*^ serving as the SD via acquiring early XY-specific high expression for testicular differentiation by itself^25^. On the other hand, other autosomal genes can be recruited as new SDs to initiate *gsdf* expression, which is exactly what has happened in medaka and its close relative *O. dancena*, where the sex determiner *sox3*^*Y*^ functions early in XY embryos to induce *gsdf* expression^54^. However, it remains to be determined whether *gsdf* is the male sex initiator and activated by *sox3*^*Y*^ directly or indirectly in this species. Therefore, an autosomal sex initiator like *gsdf* may play a critical role in the evolution of SDs and sex chromosomes.

## Methods

### Fish and chemicals

Fish work was performed in strict compliance with the recommendations in the Guide for the Care and Use of Laboratory Animals of the National Advisory Committee for Laboratory Animal Research in Singapore and approved by this committee (Permit Number: 27/09). The medaka strain *Hd-rR* and *Vg* were maintained under an artificial photoperiod of 14-h light to 10-h darkness at 26~28 °C as described^55^. Medaka embryos were maintained at 27 ± 2 °C and staged as described^56^. Chemicals were purchased from Sigma, enzymes were from New England Biolabs, and PCR reagents were from TaKaRa unless otherwise indicated.

### Plasmids

Plasmid pGsdf2Agfp expressing a chimeric mRNA between medaka *gsdf* and *gfp* was constructed, which predicts a nascent fusion protein that is self-cleaved by 2A sequence into Gsdf and GFP. Vectors pZN1gsdf and pZN2gsdf expressing ZFNs for targeting the medaka *gsdf* gene were described previously^27^. Plasmid pDmy:cherry expressing a fusion between Dmy and Cherry was constructed by inserting *dmy* coding sequence with *Bam*HI and *Eco*RI into the pCS2cherryHis backbone. Plasmids for synthesizing riboprobes were constructed by TA-cloning of PCR products into pGEM-T Easy vectors (Promega). Recombinant plasmids were sequenced on the 3130XL Sequencer (Applied Biosystems). Sequence analyses were run on Vector NTI and DNAman software.

### Microinjection

Microinjection of DNA and RNA into 1-cell stage embryos was done as previously described^36^. Microinjection of synthetic mRNAs for ZFNs into medaka embryos was described^27^. The operated embryos were reared to adulthood.

### Fish sexing

Phenotypic sex was judged based on the secondary sex characteristics, especially the shapes of the dorsal and anal fins in medaka adults^26^. Genotypic sex (XX or XY) was determined by PCR genotyping with the primer set, PG17.5 and PG17.6 amplifying *dmy* and *dmrt1* transcripts as described previously^12^.

### PCR analyses

Isolation of genomic DNA and total RNA were done as described^9^,^27^. Primers used for PCR are listed in Supplementary Table 1. Genotyping PCR was run in a 25-μl volume containing 50 ng of genomic DNA and genotyping primers for 35 cycles (94 °C for 15 s, 60 °C for 15 s and 72 °C for 30 s). RT-PCR was done as described^9^. PCR products were then separated on 1% agarose gels or 8% polyacrylamide gels and documented on a bio-imaging system (VilberLourmat).

RNA quantification was performed on the QX200 automated Droplet Digital PCR system (Bio-rad, Hercules, CA). Briefly, cDNA templates were first mixed with 2× QX200 ddPCR EvaGreen Supermix to 20-μl volume. The mixture was transferred to a 96-well plate (Eppendorf) and subjected to oil droplets generation on the automated generator (Bio-Rad, Hercules, CA). PCR in oil droplets was run for 40 cycles of 94 ^o^C for 30 s and 60 °C for 1 min. Droplets were read by QX200 Droplet Reader (Bio-Rad, Hercules, CA) and analyzed with the QuantaSoft software (Bio-Rad, Hercules, CA) that determines concentration of target cDNA as copies per microliter (copies/μl) from the fraction of positive droplets using Poisson statistics^57^. The copy number of each mRNA was further normalized with *β-actin*.

### Fluorescence *in situ* hybridization

Riboprobe synthesis, cryosectioning and fluorescence *in situ* hybridization were performed as described previously^58^. The *vasa*, *foxl2* probes were labeled with fluorescein isothiocyanate (FITC) and *gsdf*, *dmrt1* probes with digoxigenin (DIG) (Roche, Germany). Horseradish peroxidase-conjugated anti-FITC and anti-DIG antibodies were used for signal detection. The TSA Plus Fluorescein/TMR system was used (PerkinElmer Inc., Waltham, MA) to amplify the fluorescence signals.

### Immunofluorescence

Immunostaining and nuclear staining with DAPI on gonadal cryosections were done as described^31^. A monoclonal mouse anti-Gsdf antibody (αGsdf; generated by using peptide EEPAASPAST) and a polyclonal rabbit anti-Vasa primary antibody (αVasa)^59^ were used as primary antibodies at a 1:200 dilution. Goat anti-mouse antibody (Alexa Fluor 488, abcam) and anti-rabbit secondary antibody (Alexa Fluor 543, abcam) were used as secondary antibodies at a 1:200 dilution. Slides were mounted with Gold Antifade Reagent (Invitrogen) for microscopy.

### *In vivo* chromatin precipitation

Binding sites for transcription factors were predicted using the matrix^60^, and the Regulatory Sequence Analysis Tools portal (http://rsat.ulb.ac.be/rsat/, last accessed March 15, 2015). Testis samples (20 mg) from dmrt1bY:GFP transgenic fish and GFP antibody (Upstate) were used for *in vivo* chromatin immunoprecipitation (ChIP) by using the EpiQuik Tissue Chromatin Immunoprecipitation kit (Epigentek)^16^,^61^. After tissue disaggregation and cell re-suspension, DNA was sheared by sonication (9 pulses of 10s with an amplitude of 10%)^16^. ChIP procedure and analysis of DNA enrichment by real-time PCR were as described^16^,^62^.

### Cell transfection

Medaka embryonic stem cell line MES1 and spermatogonial cell line SG3 were maintained as described^8^,^39^. Cell transfection was performed by using the *Trans*IT-X2 Dynamic Delivery System (Mirus). Cells were transfected at a density of ~80% confluence and propagated or analyzed at 24–72 h post transfection.

### Microscopy

Adult fish were observed with stereomicroscope (M205FA, Leica) and photographed with the Evolution VF digital camera (MediaCybernetics) and digital camera SELP1650 (SONY). Slides were observed and photographed with the LSM 510 Meta confocal microscope (ZEISS). Cultured cells were observed with Axiovert upright microscope (Zeiss) and photographed with the AxioCam digital camera (Zeiss).

### RNA sequencing and bioinformatics

RNA samples from adult gonads were sequenced as 2 × 100-bp paired-end reads on the HiSeq 2000 platform (Illumina) by the custom service provider AITbiotech PTE LTD (Singapore). Clean reads were mapped to the medaka genome (http://www.ensembl.org/index.html) by using the reads mapper Tophat2 (version 2.0.13, http://ccb.jhu.edu/software/tophat/). Aligned sequences were assembled into transcripts by using Cufflinks (version 2.2.0, http://cole-trapnell-lab.github.io/cufflinks/cufflinks/). Cuffmerge (http://cole-trapnell-lab.github.io/cufflinks/cuffmerge/) was used to merge assemblies into a master transcriptome for analyzing differentially expressed genes (DEGs). Htseq-count^63^ was used to count the transcripts in the final transcriptome assembly as measurement of relative expression levels. Venn diagram was plotted against the reads of unigenes across samples. Count-table was passed to DESeq2^64^ to perform DEG analysis. A cutoff of FDR < 0.1 (Benjamini Hochberg method) was for MA-plots comparisons. Red dots represent statistically significant DEGs. A heat map was plotted for 400 top DEGs by sample clustering and visualization.

### Statistics

Statistical analyses were calculated by using GraphPad Prism version 6.01. Data consolidated were presented as mean ± s. e. m. or s. d. and p values were calculated by using non-parametric student’s t-test.

## Additional Information

**How to cite this article**: Zhang, X. *et al.* Autosomal *gsdf* acts as a male sex initiator in the fish medaka. *Sci. Rep.* **6**, 19738; doi: 10.1038/srep19738 (2016).

## Supplementary Material

Supplementary Information

## Figures and Tables

**Table 1:** Generation and phenotype of *gsdf* transgenic medaka^1^.

F0	Eggs	Hatched	Adults					
	174	115	TG–		TG+			
			XY♂	XX♀	XY♂	XX♀	XX♂	
							Fertile	Sterile
			37	54	8	4	3	2
F1 (XX♂× XX♀)	Founders	Progeny	XX♀		**XX**♂			
	1	131	83		**48**			
	2	60	46		**14**			
	3	28	22		**6**			
	Total	219	151		**68**			

^1^TG+, with *gsdf* transgene; TG–, without *gsdf* transgene; Masculinized fish are highlighted in bold.

**Table 2:** Generation and phenotype of *gsdf* mutant medaka^1^.

	Fish examined	*gsdf*^+/−^		*gsdf*^*−/−*^	
		XX♀	XY♂	XX♀	XY♂
F1	80	3	2	0	0
F2	120	25	37	10	**13**
F3	223	49	80	19	**37**
Total	423	77	119	29	**50**
		196		79	

^1^Feminized fish are highlighted in bold.
