# Supplementary material for: Autosomal gsdf acts as a male sex initiator in the fish medaka
Source: Sci Rep. 2016 Jan 27;6:19738. doi: 10.1038/srep19738 (PMC4728440; doi:10.1038/srep19738)
Supplement: Supplementary Information [file srep19738-s1.pdf]

Autosomal *gsdf* acts as a male sex initiator in the fish medaka

Xi Zhang[1, *], Guijun Guan[2, *], Mingyou Li[2], Feng Zhu[1], Qizhi Liu[1], Kiyoshi Naruse[3], Amaury Herpin[4], Yoshitaka Nagahama[5], Jiale Li[2, **], Yunhan Hong[1, **]

[1]Department of Biological Sciences, National University of Singapore, 14 Science Drive 4, Singapore 117543, Singapore; [2]Key Laboratory of Exploration and Utilization of Aquatic Genetic Resources of Ministry of Education and College of Fisheries and Life Sciences, Shanghai Ocean University, Shanghai 201306, China; [3]Laboratory of BioResource, National Institute for Basic Biology, Okazaki, Aichi 444-8585, Japan; [4]INRA, UR1037 Fish Physiology and Genomics, Rennes F-35000, France; [5]South Ehime Fisheries Research Center, Ehime University, Matsuyama 790-8577, Japan

*These authors contributed equally to this work.

****Correspondence:** Prof. Yunhan Hong (Email: dbshyh@nus.edu.sg)
Department of Biological Sciences,
National University of Singapore,
14 Science Drive 4, Singapore 117543
Fax: +65 6779 2486; Tel: +65 6516 2915
Prof. Jiale Li (Email: jlli@shou.edu.cn)
Key Laboratory of Exploration and Utilization of
Aquatic Genetic Resources of Ministry of Education,
Shanghai Ocean University,
Shanghai 201306, China

Running title: **Autosomal *gsdf* is a male sex initiator**

Key words: gsdf; sex determination; sex determiner; sex initiator; sex maintainer; testicular differentiation

Abbreviations: ddPCR, droplet digital PCR; DEG, differentially expressed genes; dpf, days post fertilization; FISH, fluorescence *in situ* hybridization; IF, immunofluorescence; gsdf, gonadal soma derived factor; SD, sex determiner; ZFN, zinc finger nuclease.

Supplementary materials

Supplementary Table 1 | Primer sequences.

Supplementary Figure 1 | Identification and validation of ZFN-mediated *gsdf* disruption.

Supplementary Figure 2 | Controls of FISH analysis.

Supplementary Figure 3 | Establishment of *vasa-gfp* transgenic *gsdf* knockout family.

Supplementary Figure 4 | Cell transfection assay.

Supplementary Table 1 | Primer sequences.

Primer		Sequence
Genotyping forward		TCGCCACTCCTTTCTCCTCC
Genotyping reverse		CTATGACCAGCATTTGCAGAGTTAGC
RT-PCR	actinF	CATGTGCAAAGCCGGATTCG
	actinR	GGCTTCATCTCCTACGTAGC
	gsdfF	ATGTCTTTGGCACTCATTGTCT
	gsdfR	CAATGACTCATCCTGACACCTG
	vasaF	GATTTCCGCTCAGGCAAGTG
	vasaR	GTCAATGGTGTTGGGCAGGT
	cyp19a1F	TGTCTGGTGGCAGAGCTAGT
	cyp19a1R	AACAGAAAGAAGGTCCAGGG
	foxl2F	GGTGGAACGGACTTATTTGGTTT
	foxl2R	GGGCTTTGGTAAGTGGCCAT
	dmyF	ACCCTGACCTACCGCTCCAT
	dmyR	CGCAGCTTTTCCTCATTTGG
	sdgcF	AGAACAGCGTAAGGCTTCCA
	sdgcR	GCCCATAAACCATGATGAGG
	dmrt1F	TCCGGCTCCACAGCGGTC
	dmrt1R	CAGACAGAGGGTTGGGG
	sox9bF	TTGGCCAGACAGCCAATGTT
	sox9bR	TCTCTGTTGACCCTGTTGGCTT
Real-time PCR	GsdfTrg#1Fw01	ACTGGTTACGAAACAATTAATGAT
	GsdfTrg#1Rv01	GGAATAGCAGCTCAAATGCTCAG
	GsdfTrg#2Fw01	GGTTCATATTTGTCATATATGT
	GsdfTrg#2Rv01	CTATGCTGTCATCTTATTGAGATG
	ActinFw	GAGACCTTCAACAGCCCTGC
	ActinRv	CGCTCCGTCAGGATCTTCATG
	BSF-Fw	CATTGCAGTTAAGTCCAAGCAAGC
	BSF-Rv	CTGTCCTGCAGATGTCATGGAAG

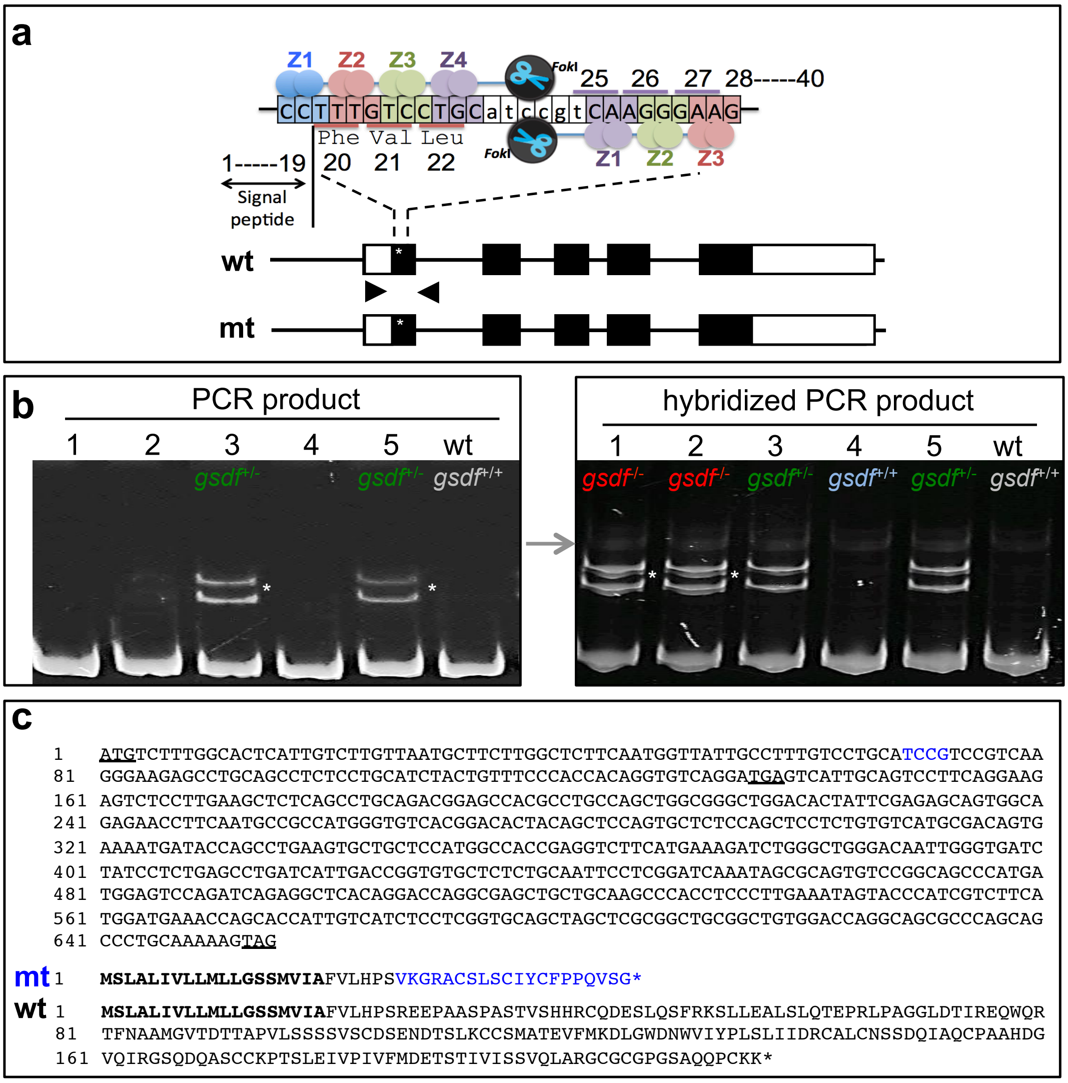

Supplementary Figure 1 | Identification and validation of ZFN-mediated *gsdf* disruption. a, Diagram showing ZFN target site. The ZFN target site (asterisk) is located into the first exon of the wildtype (wt) *gsdf* locus. Arrowheads indicate the positions and extension directions of the genotyping PCR primers. wt, wildtype; mt, mutant. **b,** Genotyping with PAGE. In *gsdf*$^{+/-}$ samples (green), distinct heteroduplex bands (asterisk) are shown on top of the homoduplex bands (Top). After denaturation and re-annealed with wt alleles, the hybridized PCR products are visualized on the second PAGE gel (Bottom). The *gsdf*$^{-/-}$ samples (red) are distinguished from the wt sample (blue). **c,** *gsdf* cDNA and protein sequence. Shown are the cDNA from the disrupted mutant (mt) *gsdf* allele (top), highlighting the 4-nt insertion (blue), authentic start and stop codons (underlined) and a new stop codon (bold) from the frame-shift mutation. The predicted mt protein is compared with the wt counterpart at the bottom, with the signal peptide in bold, and irrelevant amino acids from the truncated translation shown in blue.

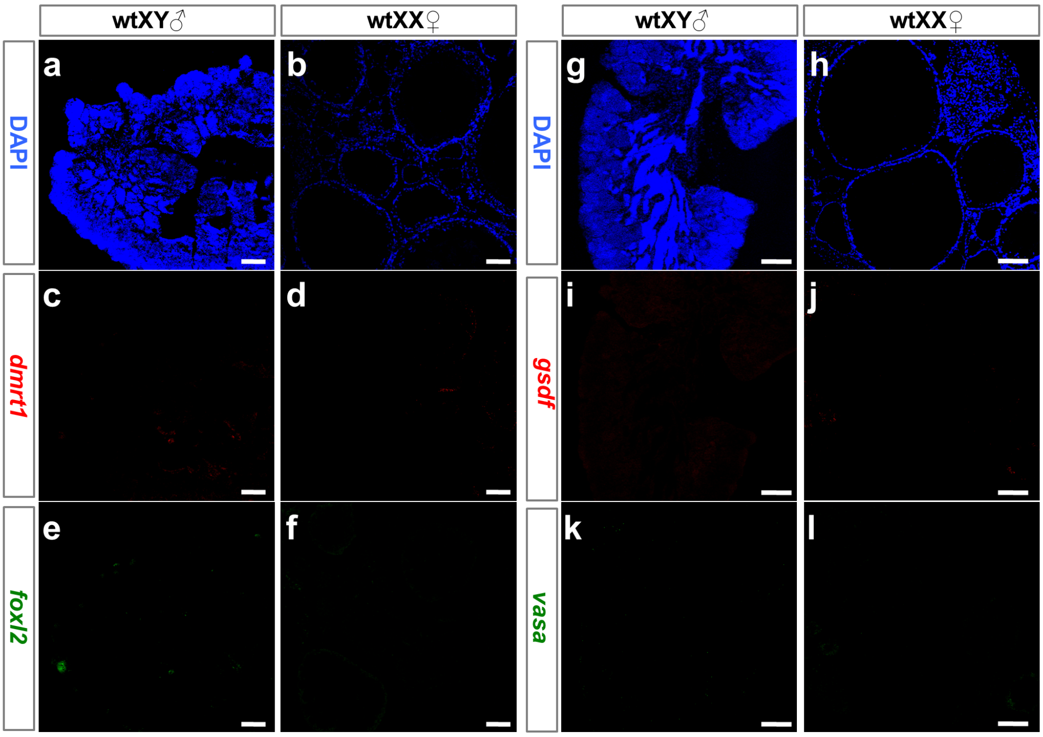

Supplementary Figure 2 | Controls of FISH analysis. Cryosections of adult gonads were subjected for FISH with sense riboprobes of *dmrt1* plus *foxl2*, *gsdf* plus *vasa* and analyzed by fluorescence microscopy. Scale bar, 100 μm.

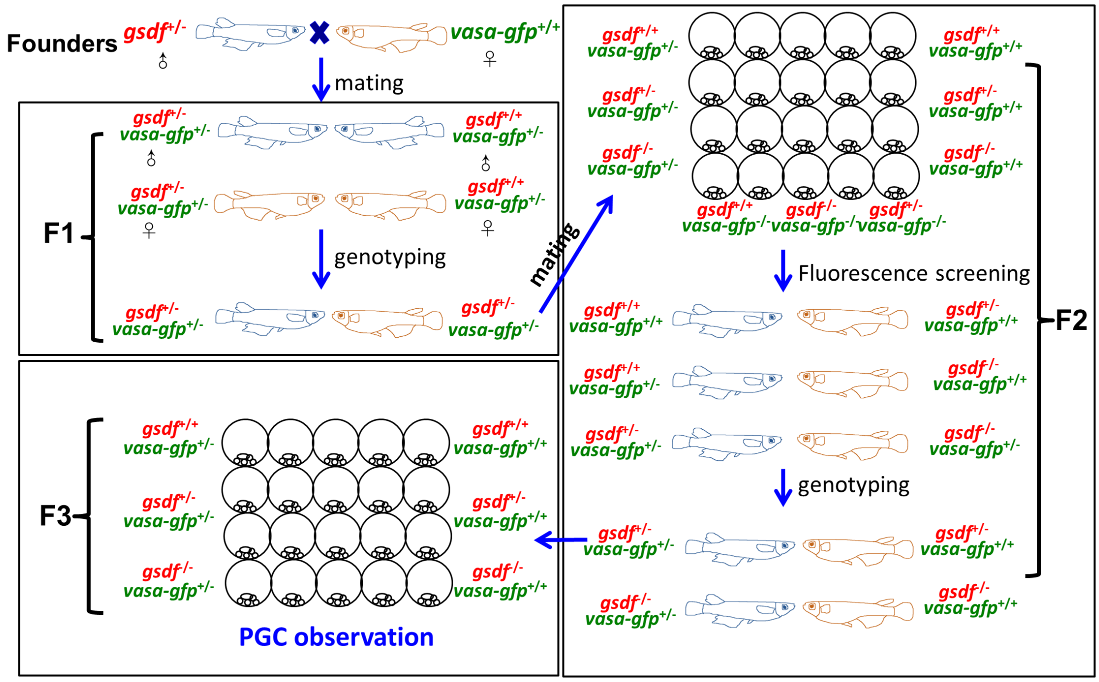

Supplementary Figure 3 | Establishment of *vasa-gfp* transgenic *gsdf* knockout family. One *gsdf*$^{+/-}$ male was mated with a *vasa-gfp* transgenic wildtype female. The F1 fish, all carrying one *vasa-gfp* allele, were genotyped. Heterozygous *gsdf*$^{+/-}$ fish were selected and mated with each other. The resulted embryos (F2) were further screened and raised till adults. These adults were further mated and the embryos (F3) were used for germ cell analysis.

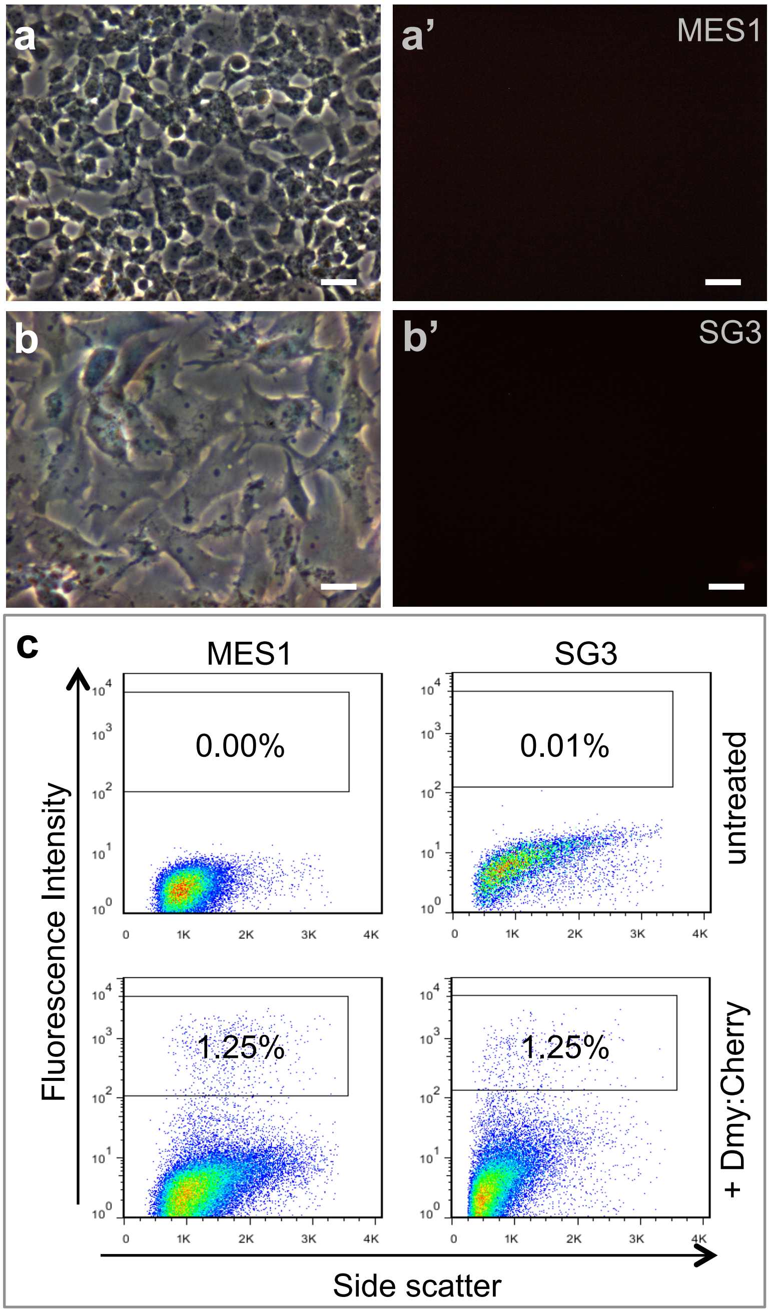

Supplementary Figure 4 | Cell transfection assay. a-b, Morphology of medaka cell lines MES1 (a, a’) and SG3 (b, b’). Scale bar, 20 µm. **c,** Dmy-expressing MES1 and SG3 cells, showing flow cytometry profile of pDmy:cherry-transfected cells on the basis of red fluorescence.
